# Using Polymerization‐Induced Self‐Assembly in Orthogonally‐Reactive Dispersants to Design Tough Nanostructured Materials: Application to Epoxy Networks

**DOI:** 10.1002/anie.202518861

**Published:** 2025-11-10

**Authors:** Theophile Ienn, Cindy Lo Van, Raphael Brunel, David Albertini, Eloi Chamczyk, Pierre Alcouffe, Guillaume Sudre, Frederic Lortie, Julien Bernard

**Affiliations:** ^1^ Université Claude Bernard Lyon 1, INSA Lyon, Université Jean Monnet, CNRS UMR 5223 Ingénierie des Matériaux Polymères F‐69621 Villeurbanne Cédex France; ^2^ CNRS, INSA Lyon, Ecole Centrale de Lyon, Université Claude Bernard Lyon 1 CPE Lyon, INL, UMR 5270 Villeurbanne 69622 France

**Keywords:** Dispersion polymerization, Nanostructured epoxy networks, PISA, Sustainability, Toughening

## Abstract

We report here how the polymerization‐induced self‐assembly approach can be implemented in orthogonally reactive dispersants to afford the preparation of nanostructured polymer networks. Precisely‐defined PMMA*‐b‐*PLMA block copolymers self‐assembling into different morphologies are first obtained in epoxy monomers through RAFT dispersion polymerization. The subsequent polymerization of the epoxide (through step growth or chain growth polymerization) affords the straightforward preparation of epoxy networks that integrate diverse BCP structures and exhibit significantly enhanced fracture toughness at extremely low block copolymer content (1% w/w).

## Graphical abstract

Polymerization‐induced self‐assembly (PISA) can be efficiently performed in orthogonally reactive dispersants such as epoxides (DGEBA or ECC) to generate a range of reactive block copolymer dispersions displaying different morphologies. Subsequent polymerization of the epoxide dispersant (through epoxy‐amine reaction or cationic photopolymerization) affords the preparation of (nano)structured epoxy networks with enhanced toughness.

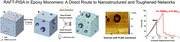

## Introduction

The depletion of natural resources and the environmental crisis caused by plastics accumulation urge us to develop polymer materials with longer functional lifetimes while minimizing the amount of material used and improving recyclability. Thermosets such as epoxy networks play a major role in modern economies with a broad range of applications, that is, coatings, automotive components, electronics, or adhesives.^[^ ^1^ ^]^ However, these materials exhibit a highly cross‐linked structure which leads to brittleness and makes them difficult to recycle. Therefore, enhancing the damage tolerance of epoxy thermosets to increase their durability is crucial. Similar to other brittle polymers, epoxy thermoset toughening is traditionally achieved through the inclusion of immiscible rubbery domains in the epoxy matrix, for example, carboxyl‐terminated butadiene–acrylonitrile liquid rubber or core‐shell rubber microparticles,^[^ ^2^ ^]^ to favor energy dissipation through cavitation and subsequent growth of the resulting voids. However, these approaches suffer from a noticeable decrease in glass transition temperature (*T*_g_), a loss of stiffness, hardness, and transparency. Mulhaupt,^[^ ^3^ ^]^ followed by Bates and Hillmyer,^[^ ^4^ ^]^ Pascault,^[^ ^5^ ^]^ and Tournilhac,^[^ ^6^ ^]^ proposed to toughen epoxy materials through the incorporation of preformed amphiphilic block copolymers (BCP) able to self‐assemble into precisely‐defined micro‐ or nanostructures in the epoxy resin. Structuring epoxy thermosets at the nanoscale then relies on the use of BCPs having one or several block(s) immiscible in the epoxy precursor and at least one block that remains miscible upon the construction of the epoxy network up to high conversions. Alternatively, nanostructures have been obtained through reaction‐induced microphase separation where the epoxy precursor is initially a good solvent of all blocks and at least one block micro‐phase separates during the polymerization.^[^ ^7^ ^]^ Both approaches have been shown to improve the mechanical properties of epoxy thermosets while limiting plasticization. Several studies highlighted that the BCP morphology in epoxy networks has a great influence on toughening, as vesicles,^[^ ^8^, ^9^ ^]^ wormlike,^[^ ^9^, ^10^ ^]^ or branched wormlike micelles^[^ ^11^ ^]^ demonstrated superior toughening in comparison with spherical micelles. The morphological control of BCP/epoxy thermosets is thus of key importance. That said, for a given BCP, the structuration within the network may be different depending on the concentration in BCP, the presence of a catalyst,^[^ ^12^ ^]^ the nature of co‐reactants (such as amine hardeners in step growth polymerization processes), or the curing cycle temperatures making robust access to the targeted morphology (in the final network) challenging.^[^ ^13^ ^]^ Besides, some limitations still need to be overcome to envision further developments for this approach: i) the toughening of the network is highly dependent on the BCP morphology and controlled access to the desirable morphologies remains delicate, ii) the incorporation of BCPs improves the toughness but sometimes lowers the *T*_g_ and the modulus of the final material making access to most suited BCP morphologies even more valuable to minimize the BCP loading, and iii) the slow dissolution of BCPs in epoxy monomers generates processing issues.

**Figure 1:**
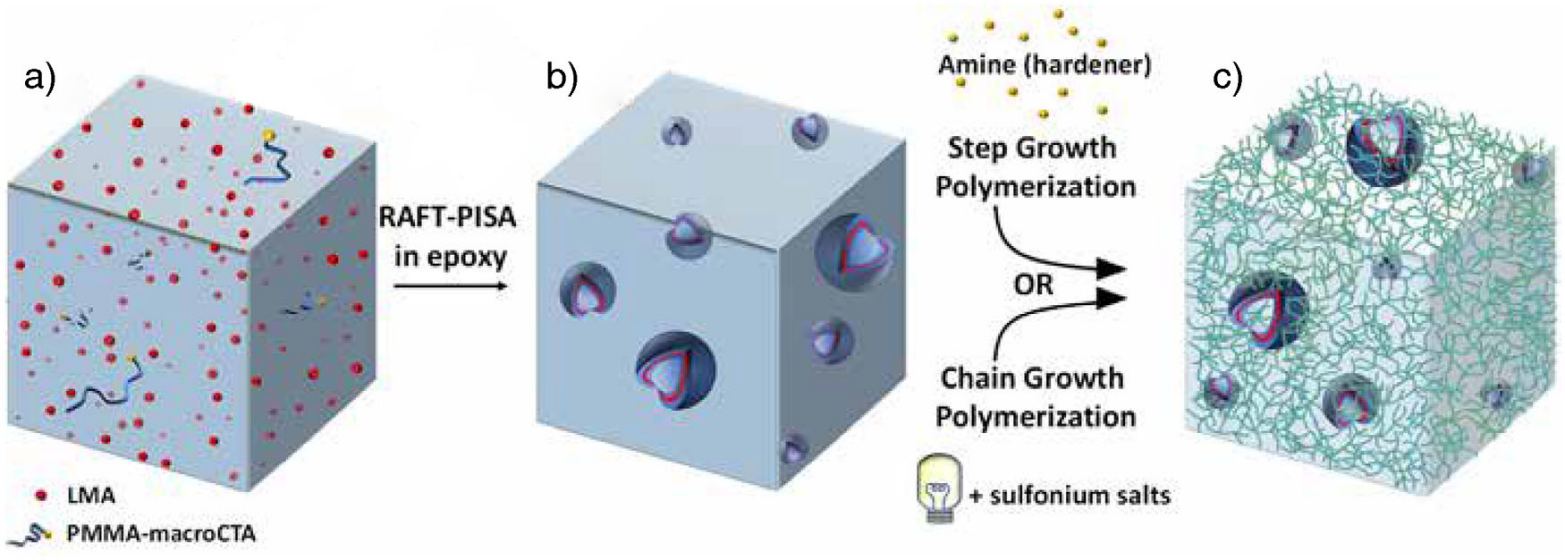
Preparation of tough (nano)structured epoxy‐amine or epoxy networks through i) RAFT‐PISA dispersion polymerization of methacrylates in epoxy monomers (steps a and b) and ii) step growth polymerization (epoxy‐amine reaction) or chain growth polymerization (cationic polymerization initiated by sulfonium salts) (step c). For the sake of simplicity, a general representation of the polymer network is given. However, note that the topology of the epoxy network is different whether it is synthesized by chain growth or step growth polymerizations (the latter leading to the formation of less heterogeneous networks than the former).

In this context, we report here a simple and general approach to produce tough nanostructured networks directly from reactive BCP epoxy dispersions generated by polymerization‐induced self‐assembly (PISA). Recent advances in reversible deactivation radical polymerizations have made PISA an ideal technique for preparing a variety of BCP morphologies such as spheres, vesicles, or worms at high concentrations.^[^ ^14^, ^15^ ^]^ In this paper, we propose to extend the general scope of the PISA process to orthogonally reactive dispersants capable of undergoing polymerization in a subsequent step. This strategy allows to generate polymer networks that integrate diverse BCP nanostructures with precisely defined dimensions and morphology (see Figure 1). As previously discussed, various preformed BCPs have been incorporated into epoxy precursors to create nanostructured thermosets. However, to our knowledge, no attention has been given so far to toughening thermosets through in situ formation of reinforcing BCPs assemblies directly into orthogonally reactive (epoxy) precursors.

To fill this gap, we demonstrate for the first time that the merits of PISA can be utilized to prepare reactive dispersions with different BCP morphologies through RAFT dispersion polymerization of methacrylates in epoxy monomers. We show how precise engineering of BCPs can be employed not only to control their self‐assembly in epoxy monomers but also to maintain their morphological stability. Finally, the reactive BCP dispersions are engaged in polyaddition (epoxy–diamine systems) or cationic UV photopolymerization to fabricate a range of nanostructured epoxy networks with enhanced toughness.

## Results and Discussion

Poly (methyl methacrylate) (PMMA) has been previously used as a stabilizing block for BCPs that self‐assemble at the nanoscale into diverse epoxy systems.^[^ ^5^ ^]^ Based on this, we prepared an initial series of PMMA macro‐chain transfer agents (macro‐CTA) with degrees of polymerization ranging from 15 to 50 and dispersities below 1.20 by bulk RAFT polymerization at 70 °C. The PMMA macro‐CTAs were subsequently chain‐extended with lauryl methacrylate (LMA), a precursor of a rubbery epoxyphobic PLMA block (*T*_g_ = ‐58 °C), by dispersion polymerization in bisphenol A diglycidyl ether (DGEBA), a widely used epoxy monomer. RAFT dispersion polymerizations of LMA were conducted in the epoxy medium (+ 4% w/w of toluene) at 70 °C for 24 h ([macro‐CTA]/[AIBN] = 3) using the methacrylic macro‐CTAs described above to prepare a series of PMMA_x_‐*b‐*PLMA_y_ block copolymer dispersions at 10 and 40% w/w solids contents (See Table S1).

**Figure 2:**
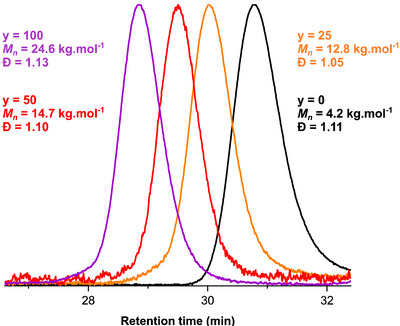
SEC traces of the PMMA_50_ *‐b*‐PLMA_y_ BCPs generated by RAFT‐PISA at 40% w/w solids content in DGEBA (from right to left, *y* = 0, 25, 50, and 100).

Macroscopically homogeneous BCP dispersions were prepared when the epoxyphobic/epoxyphilic molar ratio was typically maintained below 3 within the block copolymers. ^1^H NMR analyses demonstrated that quantitative conversion in LMA (>99%) is achieved in all cases after 24 h when the solids content is 40% w/w. In contrast, at lower solids content, typically 10% w/w solids content and high [LMA]/[macro‐CTA] ratio (100 or above), some chain extensions fail at reaching full conversion in monomer after 24 h. For example, 95% conversion is observed for a polymerization carried out with [LMA]/[PMMA_50_]/[AIBN] = 100/1/0.33 in DGEBA, (see entry 24 in Table S1) whereas quantitative conversion is achieved at 40% w/w after 12 h (see entry 23 in Table S1 and Figure S1). Conversion versus time plots confirmed that the dispersion RAFT polymerization of LMA proceeds with pseudo‐first order kinetics in the epoxy medium (Figure S1). SEC curves of PMMA_50_ *‐b*‐PLMA_y_ obtained at 40% w/w solids content in DGEBA are given in Figure 2 (chain extension experiments at 10% w/w solids content or mediated by other PMMA macroCTAs can be found in ESI, Figures S2–S4).

Consistent with an excellent blocking efficiency, no unreacted macroCTA is detected on the chromatograms after chain extension. The traces of the block copolymers shift toward higher molar masses with increasing values of *y* and show narrow molar mass distributions (Ð <1.20) confirming a good control of LMA polymerization in DGEBA.

To maintain the BCP morphologies generated by PISA in the fully cured epoxy network, it is necessary to ensure that the T_ODT_ (order‐disorder transition temperature) of the reactive BCP dispersion lies well above the temperature of the curing step during which epoxy‐amine gelation takes place. Indeed, once chemical gelation has occurred, no BCP reorganization or macrophase separation is possible. T_ODT_ was thus measured for the different BCP dispersions at 40% w/w through oscillatory rheology measurements (1 Hz) at 3 °C/min. In the case of PMMA_15_ *‐b‐*PLMA_y_ (y ranging from 15 to 45), T_ODT_ varies from 25 °C to 75 °C as *y* increases (see Figure S5). Extending the length of the PMMA and PLMA blocks leads to a substantial increase of the T_ODT_ (75 °C for PMMA_15_ *‐b‐*PLMA_45_ vs. 145 °C for PMMA_50_ *‐b‐*PLMA_50_ and 180 °C for PMMA_50_ *‐b‐*PLMA_100_). Given that the epoxy‐amine reaction is highly exothermic (≈ 90 kJ per mol of epoxy functions),^[^ ^16^ ^]^ making temperature control in the mold and thus conservation of the BCP self‐assembly challenging, we decided in the following to focus exclusively on PMMA_50_ *‐b‐*PLMA_y_ dispersions.

**Figure 3:**
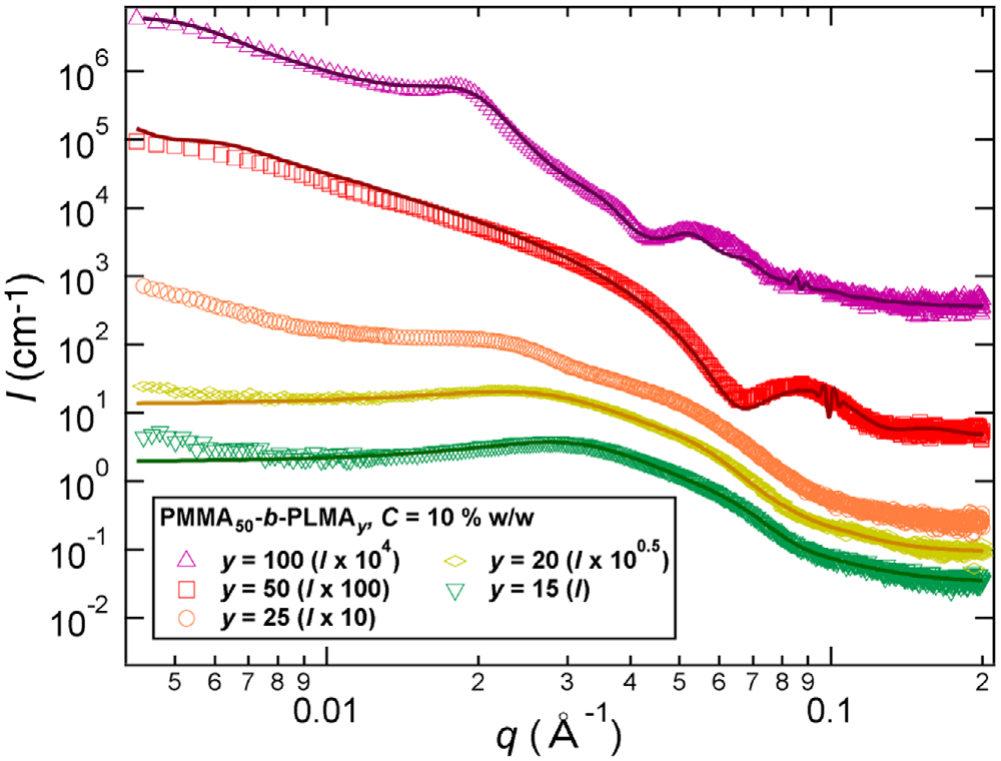
SAXS patterns of PMMA_50_ *‐b‐*PLMA_y_ dispersions synthesized at 40% w/w solids content and diluted at 10% w/w in DGEBA. Lines correspond to the data fits (*y* = 15 or 20: sphere model; *y* = 50 or 100: vesicle model).

To gain insight into the nature of the BCP morphologies obtained after RAFT‐PISA in DGEBA, we next carried out SAXS experiments on the reactive PMMA_50_ *‐b‐*PLMA_y_ dispersions. SAXS analyses of the BCP dispersions (synthesized at 40% w/w and diluted to 10% w/w in DGEBA) confirmed that the polymerization system gradually evolves from fully dissolved BCP chains to different BCP morphologies by increasing the length of the epoxyphobic PLMA block (Figure 3). The self‐assembly of the BCP was detected in DGEBA once the degree of polymerization of the PLMA block exceeded 10. When *y* values are 15 or 20, SAXS analyses highlight the formation of pure spherical morphologies with diameters ∼7–9 nm whereas when *y* = 25, a more complex SAXS pattern is observed suggesting the formation of mixed phases. Further growth of the PLMA block (*y* = 50 and 100) promotes the formation of polydisperse vesicles with diameters over 100 nm and a PLMA layer having a thickness around 9 and 15 nm, respectively. Note that several SAXS patterns exhibit a structure factor attributed to the presence of spherical micelle/vesicle aggregates. Similarly, when the PISA process was performed at 10% w/w solids content, the BCP morphology progressively shifted from tiny spheres to mixed phases and finally to large polydisperse vesicles as the length of the PLMA block increases (see Figure S6). Our attempts to image BCP dispersions using TEM and cryoTEM remained unsuccessful.

**Figure 4:**
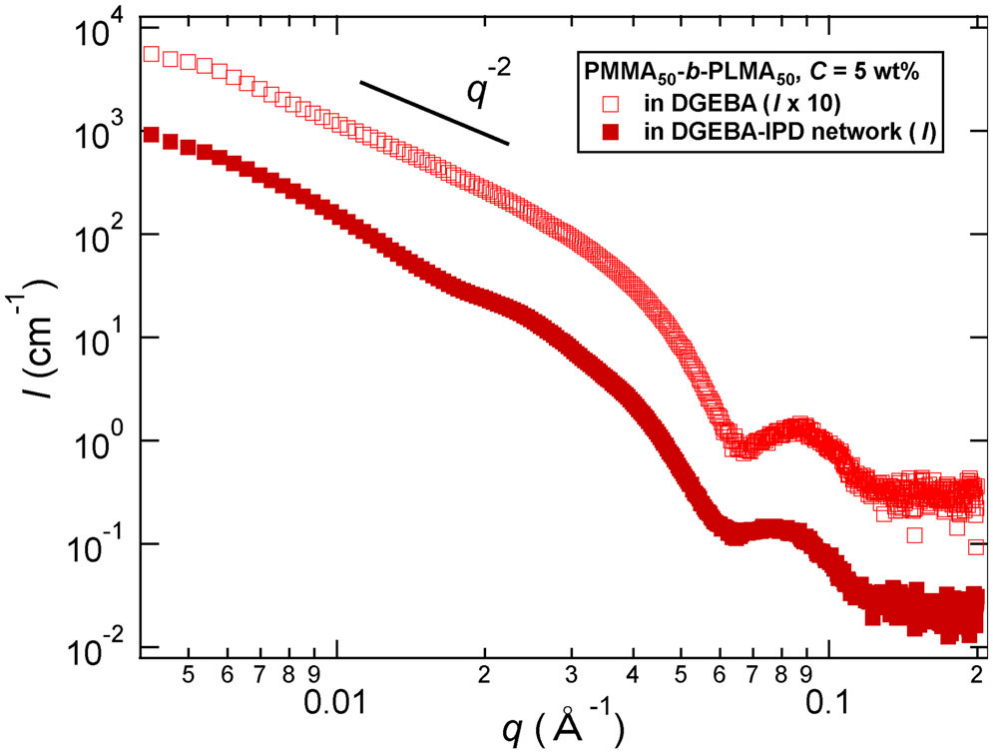
SAXS patterns of PMMA_50_‐*b*‐PLMA_50_ dispersion (generated by PISA at 40% w/w solids content) in DGEBA (diluted at 5% w/w) and of resulting DGEBA‐IPD network.

Nanostructured networks were subsequently prepared from diluted reactive dispersions to reach 1% w/w or 5% w/w PMMA_50_ *‐b‐*PLMA_y_ (*y* ranging from 15 to 100) in the final materials. DGEBA‐BCP dispersions were mixed with the hardener (isophorone diamine, IPD) for 30 min at room temperature (RT) under vacuum (to prevent the formation of bubbles in the epoxy network) and the formulations were casted in a steel mold. The dispersions were precured at RT for 24 h, 40 °C for 4 h, 80 °C for 2 h and finally post‐cured at 160 °C for 2 h. To our delight, the BCP morphologies generated in the course of the PISA process (at 40% w/w) were neither impacted by the dilution (with DGEBA) and the addition of the hardener nor by the curing cycles. A very good match between the SAXS patterns of BCP‐loaded DGEBA‐IPD networks and those of the precursor BCP dispersions was observed in agreement with the conservation of the BCP morphologies in the final materials (as exemplified in Figure 4 and Figure S7 with vesicular morphologies).

**Figure 5:**
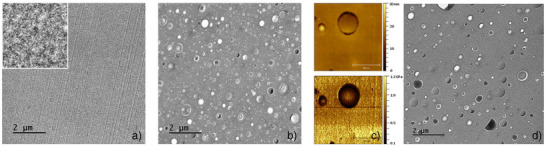
TEM and AFM pictures of BCP loaded DGEBA‐IPD networks. a) TEM picture of the 5% wt: PMMA_50_‐*b*‐PLMA_15_ loaded network (inset top left: 500 x 500 nm); b) TEM picture of 5% wt: PMMA_50_‐*b*‐PLMA_50_ loaded network c) AFM pictures (PeakForce QNM mode; top: topography; bottom: modulus) of 5% w/w PMMA_50_‐*b*‐PLMA_50_ loaded network; d) TEM picture of the network loaded with 5% wt: PMMA_50_‐*b*‐PLMA_100_.

To confirm this point, microtomed sections of the BCP‐loaded DGEBA‐IPD networks were then characterized by TEM and AFM microscopies (see images of 5% w/w BCP loaded networks from BCP dispersions generated at 40% w/w solids content in Figure 5 and 1% w/w BCP loaded networks and BCP‐free network in Figure S8). Consistent with the conclusions drawn from the SAXS studies, TEM characterization corroborated that PMMA_50_ *‐b‐*PLMA_15_ self‐assembles into tiny discrete spherical nanoparticles within the epoxy‐amine matrix (Figure 5a) and that vesicles with high dispersity in size are generated in the case of PMMA_50_ *‐b‐*PLMA_50_ and PMMA_50_ *‐b‐*PLMA_100_ block copolymers (Figures 5b–d and S9). For these two last BCP systems, AFM pictures (in PeakForce QNM mode) clearly highlight the formation of a soft layer due to the low *T*_g_ PLMA blocks, which is typical of the expected vesicular morphology (see Figures 5c, S10, and S11).

**Figure 6:**
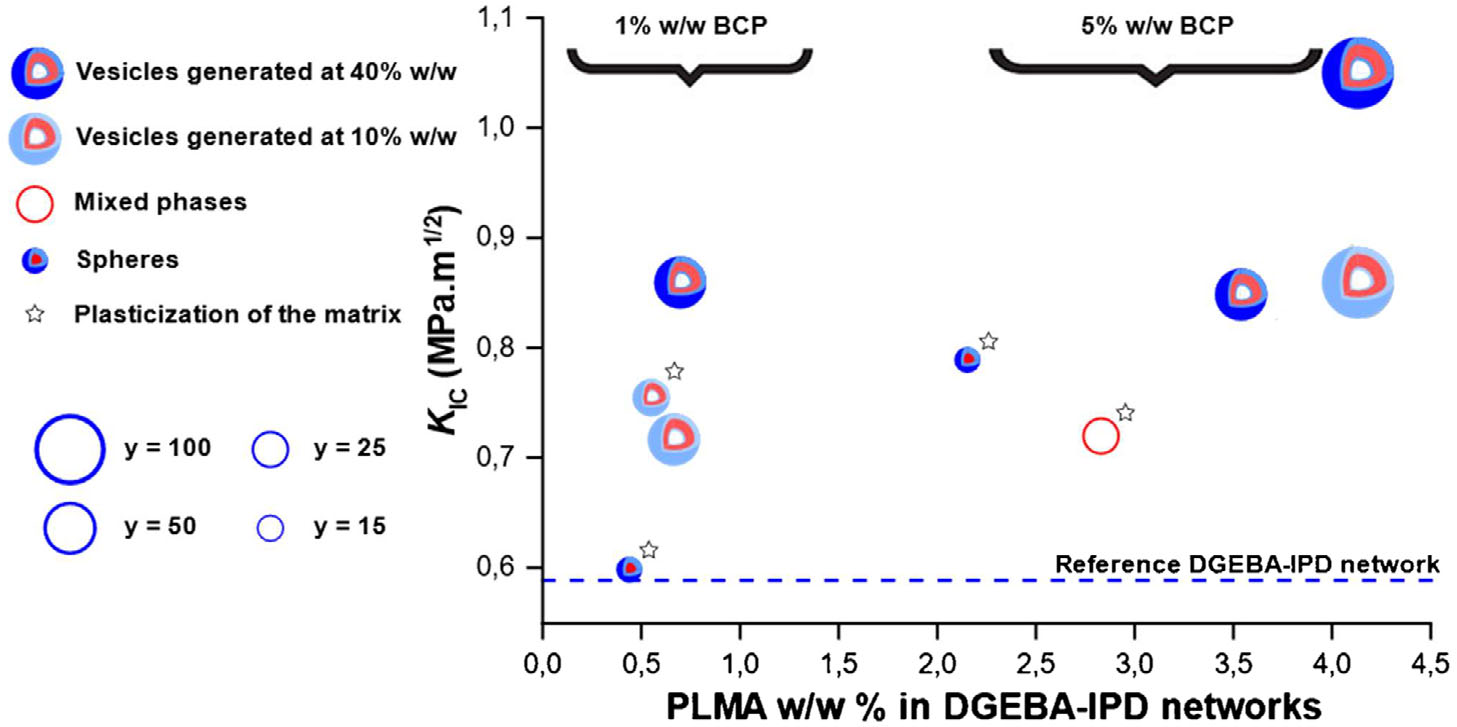
Evolution of the mode I critical stress intensity factor (*K*_IC_) as a function of the PLMA weight content in the DGEBA‐IPD network, the degree of polymerization of the PLMA block, the morphology adopted by the block copolymers in the epoxy network and the initial solids content in the PISA experiments.

To evaluate the toughness of the epoxy networks as a function of the BCP loading (0% w/w,1% w/w, or 5% w/w), composition (*y* ranging from 15 to 100) and morphology (spheres, vesicles), we next determined the mode I critical stress intensity factor (*K*_IC_) for a range of notched compact tension (CT) specimens (see Figures 6, S12, and experimental details in ESI). As anticipated, the incorporation of low *T*_g_ PLMA blocks into the epoxy network results in toughness improvement. The highest *K*_IC_ (1.05 vs. 0.59 MPa.m^1/2^ for reference DGEBA‐IPD network) is obtained with the highest PLMA content (DGEBA‐IPD network with 5% w/w of PMMA_50_ *‐b‐*PLMA_100_ initially prepared by RAFT–PISA at 40% w/w solids content). However, *K*_IC_ does not follow a monotonous evolution with the PLMA content due to the concomitant impact of the BCP morphology and of the PISA process on toughness. As shown in Figure 6, the vesicular morphologies obtained with *y* = 50 or 100 lead to significantly superior toughening as compared with spheres, without apparent plasticization of the network as confirmed by the *T*_α_ values measured by dynamic mechanical analysis (see Figure S13 and Table S2 in ESI). Relatively high *K*_IC_ values (up to 0.79 MPa.m^1/2^) can also be obtained with BCP spheres (see PMMA_50_ *‐b‐*PLMA_15_ based materials in Figure 6 and Table S2) but at the cost of a substantial drop of *T*_α_ (164 vs. 172 °C for the reference network) probably due to partial miscibility of the low molar mass BCP within the epoxy matrix. Surprisingly, for a given morphology, the conditions of RAFT‐PISA and more specifically the solids content in the precursory dispersion, have a noticeable impact on the final epoxy network toughness. Indeed, when the epoxy networks are prepared in the presence of the vesicular morphologies from PMMA_50_ *‐b‐*PLMA_50_ or PMMA_50_ *‐b‐*PLMA_100_ synthesized either at 10% w/w or 40% w/w solids content, higher *K*_IC_ values are systematically measured with the BCP dispersions generated by PISA at the highest concentration (see Figure 6). Consistent with a previous work of Bates and coworkers,^[^ ^17^ ^]^ we hypothesize that this considerable enhancement in fracture resistance stems from a significantly smaller number of sub‐100 nm vesicles and higher amounts of larger ones (100–400 nm) in the epoxy networks generated from BCP dispersions synthesized at the highest concentration (see Figure S9).

To illustrate the general applicability of our methodology, we finally investigated the PMMA‐CTA mediated RAFT dispersion polymerization of LMA in 3,4‐epoxycyclohexylmethyl 3,4‐epoxycyclohexanecarboxylate (ECC), a cycloaliphatic epoxide of interest in electronic and electrical applications. Using PMMA_50_ as macroCTA, the RAFT‐PISA process ensured the preparation of precisely‐defined BCPs (see Figure S14) adopting either spherical or vesicular morphologies as confirmed by SAXS analyses (see Figure S15). Diluted ECC dispersions exhibiting 1–5 % w/w of PMMA_50‐_ *b‐*PLMA_y_ BCPs (*y* ranging from 25 to 100) were homopolymerized under UV irradiation (365 nm, 11.4 W, 8 min) using triarylsulfonium hexafluoroantimonate as cationic photoinitiator (1% w/w) and post‐cured overnight at 100 °C to afford nanostructured epoxy networks (see Figure S16).

Consistent with the results obtained with the DGEBA/IPD system, the presence of PMMA*‐b‐*PLMA BCPs (using the PISA strategy) promoted the generation of ECC polymer networks with significantly enhanced toughness. Remarkably, the incorporation of only 1% w/w of PMMA_50_ *‐b‐*PLMA_75_ (prepared at 40% w/w solids content) induced an increase of *K*_IC_ of ∼+77% (0.69 MPa.m^1/2 ^vs. 0.39 MPa.m^1/2^ for the reference epoxy network).

## Conclusions

In summary, we have developed a new approach to designing tough (nano)structured polymer networks by implementing the PISA process in orthogonally reactive media. We first showed that RAFT dispersion polymerization of lauryl methacrylate can be efficiently performed in epoxy monomers (DGEBA and ECC) to synthesize a series of PMMA*‐b‐*PLMA block copolymer nano‐objects. We demonstrated that the degrees of polymerization for the methacrylic blocks can be adjusted (to target a high T_ODT_) so that the BCP morphologies generated by PISA are not altered by the successive processing steps. The polymerization of these reactive dispersions consequently resulted in the preparation of epoxy networks that integrate the different BCP morphologies (spheres, vesicles) initially generated by PISA. Interestingly, the incorporation of vesicular morphologies prepared by PISA at very high solids contents (40% w/w) proved particularly suitable to improve the damage tolerance of the polymer networks at very low block copolymer contents without inducing undesirable plasticization.

The simplicity and the versatility of this novel approach, which engages PISA in orthogonally reactive dispersants, opens promising new avenues for designing toughened nanostructured polymer networks.

## Supporting Information

Synthetic procedures, polymer characterization (^1^H NMR, SEC, DSC, DMA, and rheology), SAXS studies on BCP dispersions and epoxy networks, cryo‐TEM, TEM, AFM supplementary data, preparation of notched CT specimens, and determination of *K*_IC_.

## Conflict of Interests

The authors declare no conflict of interest.

## Supporting information



Supporting Information

## Data Availability

The data that support the findings of this study are available from the corresponding author upon reasonable request.
