# Supplementary material for: Using Polymerization‐Induced Self‐Assembly in Orthogonally‐Reactive Dispersants to Design Tough Nanostructured Materials: Application to Epoxy Networks
Source: Angew Chem Int Ed Engl. 2025 Nov 10;65(1):e18861. doi: 10.1002/anie.202518861 (PMC12759250; doi:10.1002/anie.202518861)
Supplement: Supplementary file 1 — Supporting Information [file ANIE-65-e18861-s001.docx]

**Using Polymerization-Induced Self-Assembly in Orthogonally-Reactive Dispersants to Design Tough Nanostructured Materials: Application to Epoxy Networks.**

Theophile Ienn, ^[a]^ Cindy Lo Van, ^[a]^ Raphael Brunel, ^[a]^ David Albertini,^[b]^ Eloi Chamczyk, ^[a]^ Pierre Alcouffe, ^[a]^ Guillaume Sudre, ^[a]^ Frederic Lortie, ^[a]^ and Julien Bernard*^[a]^

[julien.bernard@insa-lyon.fr](mailto:julien.bernard@insa-lyon.fr)

^[a]^ Universite Claude Bernard Lyon 1, INSA Lyon, Université Jean Monnet, CNRS UMR 5223, Ingénierie des Matériaux Polymères F-69621 Villeurbanne Cédex, France

^[b]^ CNRS, INSA Lyon, Ecole Centrale de Lyon, Université Claude Bernard Lyon 1, CPE Lyon, INL, UMR 5270, 69622 Villeurbanne, France.

**Table of Contents**

[**1. Experimental section** 2](#_Toc204157295)

[**2. Supplementary Data: Figures and Tables** 9](#_Toc204157296)

# **1. Experimental section**

**Sample Nomenclature:**

The copolymers are denoted using the notation ^40^_5_PMMA_x_-b-PLMA_y_, where:

- ^40^ (superscript) corresponds to the weight fraction of solids (in % w/w) used in the synthesis of the block copolymer by RAFT-PISA.
- ^5^ (subscript) refers to the final concentration (in % w/w) of the copolymer in the epoxy network.
- PMMAₓ-b-PLMAᵧ indicates the chemical structure of the diblock copolymer, with x and y representing the degrees of polymerisation (DP) of the PMMA and PLMA blocks, respectively.

For example, ^40^_5_PMMA_50_-b-PLMA_100_ refers to a block copolymer synthesized at 40% w/w solid content, then diluted to 5% w/w in the epoxy network, composed of a PMMA block with DP = 50 and a PLMA block with DP = 100.

**Materials:**

Methanol (≥ 99%) and toluene (≥ 99%) were purchased from Carlos Erba Reagents. Methyl methacrylate (MMA, 99%), lauryl methacrylate (LMA, 96%), 4-cyano-4-(phenylcarbonothioylthio)pentanoic acid (≥ 99%), aluminum oxide (standard grade, Brockmann I), azobis(isobutyronitrile) (AIBN, 98%), bisphenol A diglycidyl ether (DGEBA, DER332 grade), 3,4-epoxycyclohexanemethyl 3,4-epoxycyclohexanecarboxylate (ECC, ≥ 99%), isophoronediamine (IPD, ≥ 99%) and triarylsulfonium hexafluoroantimonate salts (TAS, 50% w/w in proprylène carbonate) were purchased from Sigma-Aldrich. Methyl methacrylate and lauryl methacrylate were purified by passing through a column packed with aluminum oxide to remove the inhibitor. All other reagents and solvents were used as received without further purification.

**Nanostructured network synthesis:**

Step 1: PMMA-macroCTA synthesis:

Methyl methacrylate (26.9 g, 2.69 $\times$ 10^-1^ mol), 4-cyano-4-(phenylcarbonothioylthio)pentanoic acid (1 g, 3.58 $\times$ 10^-3^ mol) as RAFT agent and AIBN (0.118 g, 7.18 $\times$ 10^-4^ mol) as initiator were added in a Schlenk tube. The polymerization mixture ([MMA]/[CTA]/[AIBN] = 75/1/0.2) was degassed by five consecutive freeze-pump-thaw cycles and heated to 70 °C in an oil bath. The polymerization was stopped after 3.33 hours by plunging the tube into iced water to reach a monomer conversion around 66%. The resulting polymer (PMMA) was precipitated in cold methanol, dried under vacuum, and isolated as a powder (16.3 g). The number-average molar mass (Mn) was determined by ^1^H NMR using CDCl_3_ as the solvent from the relative integration of aromatic peaks from the CTA (1H, t, 7.45 ppm) and the methoxy protons (O-CH_3_) of the PMMA repeating unit (3H, s, 3.52 ppm), and by SEC analysis in THF. *M*_n_ _NMR_= 5000 g.mol^−1^ ; *M*_n_ _SEC_ = 4200 g.mol^−1^ ; *Đ* = 1.12.

Step 2: PMMA chain extension with LMA in DGEBA:

DGEBA (14.4 g, 4.23 $\times$ 10^-2^ mol), toluene (1.04 g, 1.13 $\times$ 10^-2^ mol) and PMMA (1.65 g, 3.1 $\times$ 10^-4^ mol) were added to a 50 mL round-bottom flask and heated to 70 °C for 1 h. In parallel, AIBN (0.017 g, 1.04 $\times$ 10^-4^ mol) was dissolved in a vial containing lauryl methacrylate (7.95 g, 3.12 $\times$ 10^-2^ mol). Once complete dissolution was achieved and the reaction flask had returned to room temperature, the monomer/initiator solution was directly transferred into the flask. After five freeze–pump–thaw cycles to remove oxygen, the chain extension was carried out at 70 °C. The RAFT polymerization was stopped after 24 hours by rapid cooling in an ice bath. The number-average molar mass was determined by ^1^H NMR in toluene-*d*_6_ as the solvent, based on the relative integration of the methoxy protons (O-CH_3_) of PMMA (3H, d, 3.15 ppm) and the methylene protons (O-CH_2_) of PLMA (2H, d, 3.83 ppm). The dispersity (*Đ*) was assessed by SEC analysis in THF. *M*_n_ _NMR_ = 30700 g.mol^−1^; *M*_n_ _SEC_ = 24600 g.mol^−1^; *Đ* = 1.13.

Step 2 bis: PMMA chain extension with LMA in ECC:

ECC (4.8 g, 1.90 $\times$ 10^-2^ mol) and PMMA (0.7 g, 1.32 $\times$ 10^-4^ mol) were introduced into a 50 mL round-bottom flask and heated at 70 °C for 1h. In parallel, AIBN (0.0072 g, 4.37 $\times$ 10^-5^ mol) was dissolved in a vial containing lauryl methacrylate (2.50 g, 9.83 $\times$ 10^-3^ mol). After complete dissolution and once the flask was cooled down to room temperature, the monomer/initiator solution was added to the reaction mixture. The system was degassed by five freeze–pump–thaw cycles to remove dissolved oxygen, and the RAFT polymerization was carried out at 70 °C for 24 h. The reaction was quenched by rapid cooling in an ice bath. The number-average molar mass was determined by ^1^H NMR in toluene-d_6_, using the relative integration of the methoxy protons (O-CH_3_) of PMMA (3H, d, 3.15 ppm) and the methylene protons (O-CH_2_) of PLMA (2H, d, 3.83 ppm). The dispersity (*Đ*) was determined by SEC analysis. Mn _NMR_= 24400 g.mol^−1^; *M*_n_ _NMR_ = 18400 g.mol^−1^; *Đ* = 1.11.

Step 3: Crosslinking of the DGEBA dispersion by polyaddition:

The DGEBA-based dispersion was crosslinked via polyaddition with isophoronediamine (IPD). After completion of the RAFT-PISA process in DGEBA, the reaction mixture (13.5 g) was cooled to room temperature. Additional DGEBA (74 g) was then incorporated to adjust the overall block copolymer content to 5% w/w. The mixture was stirred under vacuum at 80 °C for 30 min. After cooling to room temperature, IPD (20.5 g; stoichiometric ratio with respect to epoxy groups) was added, and the resulting mixture was stirred under vacuum for an additional 30 min to ensure complete homogenization of the dispersion and prevent bubble formation. The formulation was then poured into a custom mold consisting of two metal plates separated by a 5 mm thick silicone spacer (see below). The formulation was then cured in a convection oven under air following a multi-step schedule: overnight at room temperature, 4 h at 40 °C, 2 h at 80 °C, and 2 h at 160 °C under air. After curing, a 120 × 120 × 5 mm³ epoxy plate was obtained and cooled to room temperature prior to characterization.


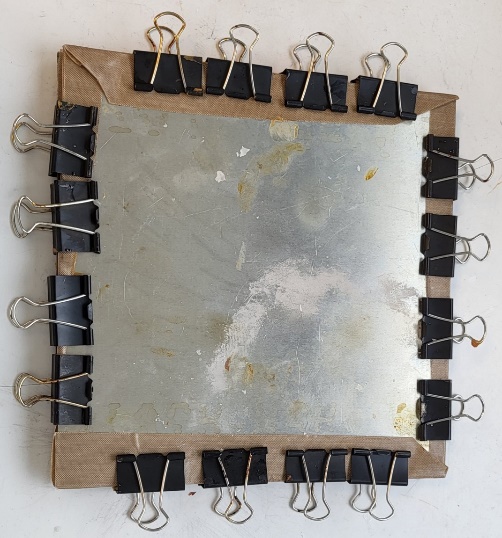


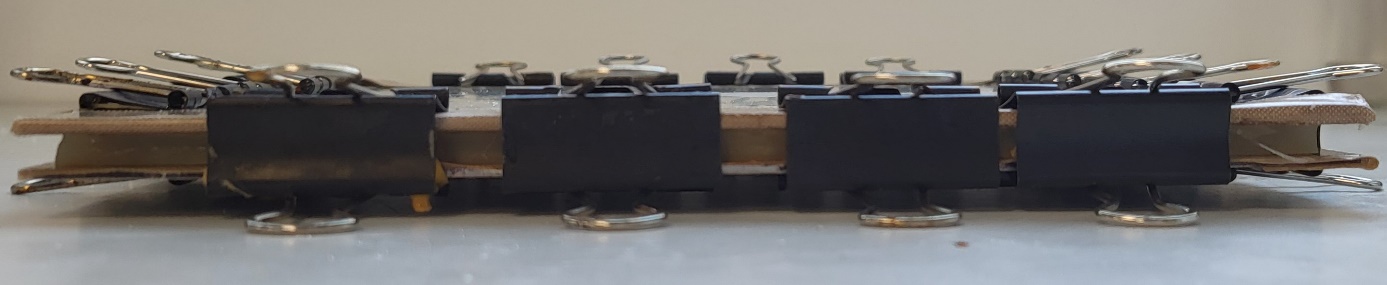


Step 3 bis: Crosslinking of the ECC BCP dispersion via cationic homopolymerization:

The ECC-based dispersion was crosslinked via cationic homopolymerization initiated by triarylsulfonium salts (TAS). After completion of the RAFT-PISA process in ECC, the reaction mixture (5 g) was cooled to room temperature. ECC (35 g) was next added to the BCP dispersion to adjust the overall block copolymer content to 5% w/w in the final formulation. The mixture was stirred under vacuum at 80 °C for 30 min. After cooling to room temperature, TAS (0.4 g; 1wt%) was added, and the mixture was stirred under vacuum for an additional 30 min to ensure full homogenization and to eliminate entrapped air. The formulation was then poured into a metal mold containing a 5 mm thick silicone frame (see below). Photopolymerization was performed using a 365 nm, 11.4 W UV lamp following a stepwise protocol: three cycles of 10 s, three cycles of 30 s, followed by multiple cycles of 1-2 min, while monitoring the surface temperature with an infrared thermal camera to ensure it remained below 90 °C. Between each cycle, a pause of 30 s to 2 min was applied to allow the surface temperature to drop below 50 °C. After UV curing, a post-curing step was conducted overnight at 100 °C in a convection oven under air. Upon demoulding, two plates of dimensions 80 x 80 x 2.5 mm^3^ were obtained and cooled to room temperature prior to characterisation.


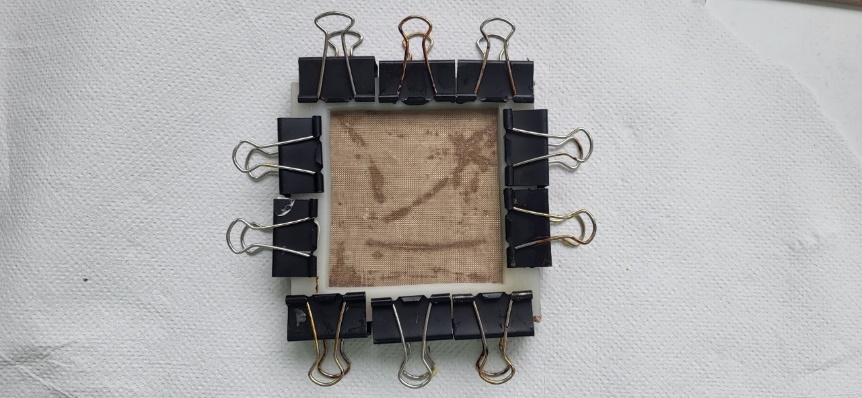


**NMR Analysis:**

^1^H NMR spectroscopy was employed to determine the monomer conversion, the degree of polymerization and the molar mass of the synthesized polymers using a Bruker Avance III 400 MHz spectrometer equipped with an autosampler. The analyses were conducted at 25 °C in CDCl_3_ (PMMA) or in toluene-*d6* (PMMA*-b-*PLMA). Chemical shifts are reported in parts per million (ppm) relative to the TMS peak at 0 ppm.

**SEC Analysis:**

Size-exclusion chromatography (SEC) was performed using a Shimadzu modular system consisting of an SIL-20AC autoinjector, an LC-20AD pump, a DGU-20A degasser, a CTO-20A column oven, a RID-10A differential refractive index detector, and a Waters WAT044228 column. Tetrahydrofuran (THF) at 40 °C was used as the mobile phase at a constant flow rate of 1 mL.min^-1^. The system was calibrated with linear poly(methyl methacrylate) standards ranging from 2 230 to 914 000 g.mol^-1^.

**Rheological Analysis:**

The order–disorder transition temperature (T_ODT_) was determined using a DHR2 stress-controlled rheometer (TA Instruments) operating in oscillatory mode with a cone–plate geometry of 40 mm diameter. Temperature control was ensured by a high-power Peltier system. Temperature sweep tests were performed on DGEBA and ECC-based dispersions containing block copolymers. A temperature ramp of 3 °C/min was applied, and the storage modulus (G′) was recorded at a fixed frequency of 1 Hz in the linear viscoelastic domain. T_ODT_ was defined as the temperature at the onset of the drop in G′, corresponding to the disruption of the ordered microstructure.

**SAXS Analysis:**

The small-angle X-ray scattering patterns of samples were measured either at ESRF on beamline D2AM (proposal A02-1-905, doi: 10.15151/ESRF-ES-1550904570) or on a Xeuss 3.0 SAXS/WAXS instrument from Xenocs. The Xeuss 3.0 was equipped with a copper source (λ = 1.542 Å) and a detector Dectris EIGER2 Si 1M. It was used in standard configurations at two sample-to-detector distances (90 cm, 180 cm). At ESRF, the wavelength was set at λ = 0.6888 Å and the 2D ImXPad D5 detector was placed at 3.012 m from the samples. The wavevector and intensity calibrations were carried out using silver behenate and a glassy carbon standard, respectively. Solid samples were directly placed on the beam path while for liquid samples, either 1-mm thick glass capillaries (WJM-Glas, for Xenocs) or 3-mm thick glass tubes (Deutero GmbH, for D2AM) were used. Scattering data were normalized by transmitted intensity, and the signal of the background was recorded and subtracted to each sample intensity. The I vs q patterns were obtained after normalization of the data by the exposure time and transmission and sample thickness, followed by azimuthal average and subtraction of the matrix/solvent.

The data were fitted with the SASView software using simple vesicular, spherical or cylindrical models; a sticky-hard-sphere structure factor was used for systems presenting structure peaks. The scattering length densities were chosen equal to 8.79 × 10^−6^ Å^−2^ for PLMA and 10.50 × 10^−6^ Å^−2^ for DGEBA and PMMA chains swollen in DGEBA; the models provided decent fits of the data and the analysis gave access to characteristic sizes/distances expected to be found in the samples.

The parameters used for our fittings and the results obtained from the fitting are given below.

**Vesicles:** Given that some of the curves obtained for vesicular morphologies show a broad structure peak, we have chosen to model these samples using a vesicle model combined with a “sticky hard-sphere” structure factor. In the vesicle model, SASview 5^1^ returns the values of the vesicle core radius, the shell thickness and their polydispersities. Regarding the structure factor, since the observed primary peak (around 2 × 10^−2^ Å^−1^) indicates a characteristic distance of around 300 Å (which is smaller than the radius of the vesicle), the reason for its presence is not obvious. We have thus chosen an empirically suited structure factor: the “sticky hard-sphere” structure factor returns a volume fraction and an effective radius, as well as a perturbation parameter and a stickiness, as reported in the following table:

| Sample | PMMA_50_*-b-*PLMA_50_, | PMMA_50_*-b-*PLMA_100_ | PMMA_50_*-b-*PLMA_100_ |
| --- | --- | --- | --- |
| wt% at synthesis | 40 | 40 | 10 |
| Radius (Å)  Polydispersity | 508.2 ± 8.2  0.402 ± 0.022 | 683.9 ± 16.8  0.696 ± 0.038 | 877.2 ± 7.8  0.44 ± 0.02 |
| Thickness (Å)  Polydispersity | 91.8 ± 2.4  0.14 ± 0.02 | 152.8 ± 6.0  0.18 ± 0.03 | 139.8 ± 3.8  0.16 ±0.02 |
| Background (× 10^2^ cm^−1^) | 4.38 ± 0.40 | 3.29 ± 0.24 | 1.71 ± 0.22 |
| Effective radius | – | 182.9 | 152.9 |
| Volume fraction | – | 0.096 | 0.098 |
| Perturbation | – | 0.040 | 0.01 |
| Stickiness | – | 0.195 | 0.107 |
| *Χ*^2^ | 0.0244 | 0.0201 | 0.124 |
| Figure | 3 | 3 | S6 |

**Spheres:** Given that all the curves obtained for spherical morphologies show a broad structure peak, we have chosen to model these samples using a core-shell sphere model combined with a hard-sphere structure factor. The SLD of the shell was maintained equal to that of the continuous medium to properly adapt the outer radius of the spheres in the hard-sphere structure factor. In SASview 5, the core-shell sphere model returns the radius of the sphere core, the thickness of the shell and their polydispersities. The hard-sphere structure factor returns solely the volume fraction. The obtained results are reported in the table below; although the errors on the fitting parameters are high, starting the fit with varying physically sound parameters always leads to the reported values.

| Polymer | PMMA_50_-*b*-PLMA_15_ | PMMA_50_*-b-*PLMA_20_ | PMMA_50_*-b-*PLMA_15_ | PMMA_50_*-b-*PLMA_20_ |
| --- | --- | --- | --- | --- |
| wt% of synthesis | 40 | 40 | 10 | 10 |
| Radius (Å)  Polydispersity | 40.7 ± 22.0  0.26 ± 0.22 | 35.1 ± 30.0  0.47 ± 0.32 | 34.8 ± 30.8  0.25 ± 0.22 | 37.3 ± 29.2  0.47 ± 0.34 |
| Thickness (Å) | 49.7 ± 20.1 | 69.2 ± 27.8 | 44.9 ± 33.5 | 73.1 ± 40.5 |
| Volume fraction | 0.136 ± 0.018 | 0.105 ± 0.011 | 0.177 ± 0.150 | 0.097 ± 0.088 |
| Background  (× 10^2^ cm^−1^) | 3.32 ± 5.47 | 2.18 ± 5.51 | 0.36 ± 0.54 | 0.23 ± 0.45 |
| *Χ*^2^ | 0.176 | 0.192 | 0.236 | 0.111 |
| Figure | 3 | 3 | S6 | S6 |

**TEM Analysis:**

The morphology of the cured epoxy networks was examined by transmission electron microscopy (TEM) using a JEOL 1400 Flash microscope operated at 120 kV. Ultrathin sections (~70 nm) were prepared at room temperature using a Leica EM UC7 ultramicrotome.

**AFM Analysis:**

All measurements were performed with a dimension Icon AFM with Nanoscope 6 electronic from Bruker using the Peak Force Tapping mode, Peak Force Quantitative Nanomecanics (QNM) and Force Volume. AFM tips are RTESPA 150-30 and were purchased from Bruker, France. Their force constant, tip radius and sensibility of 2.676 N.m-1, 31 nm and 42,77 nm/V, respectively, are used for the calculation of nanomechanical mappings including the moduli. The same tip was used throughout the measurements to ensure proper comparison of modulus mapping between samples. The peak force setpoint was set to a force of 1 nN (sometime 2 nN), the peakforce amplitude was set mainly at 20 nm, and up to a maximum of 50 nm. PeakForce frequency was 2 kHz and the scan rate was 0.5 Hz. Gwyddion 2.68 software was used for data visualization and analysis. Nanoscope Analysis 3.0 R1SR2 was used for Force Curve analysis.

**DMA Analysis:**

Dynamic mechanical analysis of the cured epoxy networks was performed in torsional mode using a strain-controlled ARES G2 rheometer (TA Instruments). Temperature sweep tests were conducted on parallelepiped samples (40 x 5 x 3 mm^3^), which had been prepared using a Charlyrobot milling machine. Measurements were carried out under air, at a heating rate of 3 °C/min, a fixed frequency of 1 Hz, and a strain amplitude of 0.05% (in the linear viscoelastic domain). The thermomechanical transition temperature (T_α_) corresponding to the main α-relaxation was determined at the maximum of tan δ peak.

**Crack propagation tests:**

Crack propagation experiments were conducted on compact tension (CT) specimens (see picture below) to determine the mode I critical stress intensity factor (*K*_IC_) in accordance with the ASTM D5045 standard. Samples were prepared from cured epoxy plates using a Charlyrobot milling machine. For DGEBA-IPD networks, the CT specimens had dimensions of 29.8 x 28 x 5 mm^3^, while for ECC-based networks, the thickness was reduced to 2.5 mm. Tests were conducted using an MTS 2/M testing machine equipped with a 1 kN load cell, at a loading rate of 1 mm/min and under controlled temperature (25°C). In a first step, the samples were cut with a band-saw to realize a sharp notch. A razor blade was then used to initiate a natural crack. Given values result from an average of data obtained on at least five specimens per formulation. The *K*_IC_ was calculated using the following equation:

$$K_{IC}=\frac{F}{B\sqrt{W}}f\left( \frac{a}{W} \right)$$

where F is the load at crack initiation, B is the specimen thickness, W is the specimen width, a is the initial crack length, and f(a/W) is the geometry-dependent shape factor (hinge factor).


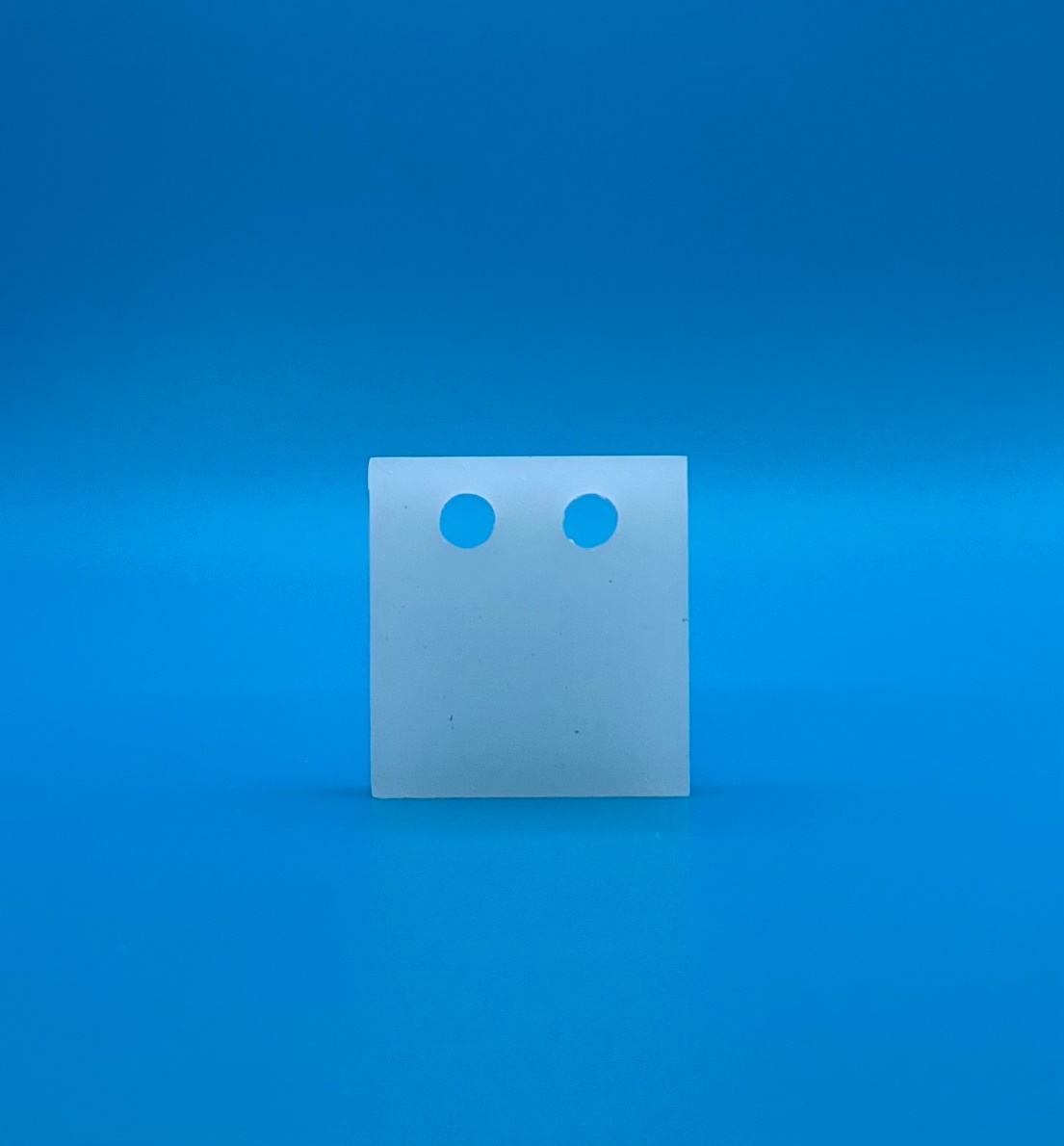


# **2. Supplementary Data: Figures and Tables**

| **No.** | **Block copolymers** | **Solid content** | **Conversion (%)** | ***M*_n_ (g.mol^-1^)** | ***M*_w_/*M*_n_** | **Macroscopic**  **appearance** | **Φ (PLMA)^a^** |
| --- | --- | --- | --- | --- | --- | --- | --- |
| 1 | PMMA_15_-*b*-PLMA_15_ | 40 | >99 | 7 500 | 1.17 | Transparent gel | 0.76 |
| 2 | PMMA_15_-*b*-PLMA_15_ | 10 | 97 | 6 600 | 1.14 | Transparent viscous liquid | 0.76 |
| 3 | PMMA_15_-*b*-PLMA_30_ | 40 | >99 | 11 000 | 1.19 | Turbid Gel | 0.87 |
| 4 | PMMA_15_-*b*-PLMA_30_ | 10 | 84 | 7 800 | 1.09 | Slightly turbid viscous liquid | 0.87 |
| 5 | PMMA_15_-*b*-PLMA_45_ | 40 | >99 | 15 800 | 1.16 | Turbid Gel | 0.91 |
| 6 | PMMA_15_-*b*-PLMA_45_ | 10 | 71 | 8 900 | 1;12 | Phase separation | 0.91 |
| 7 | PMMA_15_-*b*-PLMA_70_ | 40 | 99 | 18 600 | 1.17 | Phase separation | 0.94 |
| 8 | PMMA_25_-*b*-PLMA_30_ | 40 | >99 | 8 900 | 1.18 | Transparent gel | 0.78 |
| 9 | PMMA_25_-*b*-PLMA_65_ | 40 | >99 | 15 000 | 1.16 | Turbid Gel | 0.89 |
| 10 | PMMA_25_-*b*-PLMA_100_ | 40 | 98 | 20 500 | 1.20 | Phase separation | 0.92 |
| 11 | PMMA_50_-*b*-PLMA_10_ | 40 | >99 | 8 400 | 1.08 | Transparent gel | 0.39 |
| 12 | PMMA_50_-*b*-PLMA_10_ | 10 | >99 | 8 900 | 1.08 | Transparent liquid | 0.39 |
| 13 | PMMA_50_-*b*-PLMA_15_ | 40 | >99 | 10 600 | 1.09 | Transparent gel | 0.49 |
| 14 | PMMA_50_-*b*-PLMA_15_ | 10 | >99 | 9 700 | 1.09 | Transparent liquid | 0.49 |
| 15 | PMMA_50_-*b*-PLMA_20_ | 40 | >99 | 11 500 | 1.06 | Transparent gel | 0.57 |
| 16 | PMMA_50_-*b*-PLMA_20_ | 10 | >99 | 11 300 | 1.08 | Transparent viscous liquid | 0.57 |
| 17 | PMMA_50_-*b*-PLMA_25_ | 40 | >99 | 12 800 | 1.05 | Transparent gel | 0.62 |
| 18 | PMMA_50_-*b*-PLMA_25_ | 10 | >99 | 12 900 | 1.05 | Transparent viscous liquid | 0.62 |
| 19 | PMMA_50_-*b*-PLMA_50_ | 40 | >99 | 14 700 | 1.10 | Transparent gel | 0.76 |
| 20 | PMMA_50_-*b*-PLMA_50_ | 10 | 97 | 14 900 | 1.14 | Transparent viscous liquid | 0.76 |
| 21 | PMMA_50_-*b*-PLMA_75_ | 40 | >99 | 18 400 | 1.11 | Slightly turbid gel | 0.83 |
| 22 | PMMA_50_-*b*-PLMA_75_ | 10 | 97 | 19 300 | 1.10 | Slightly turbid viscous liquid | 0.83 |
| 23 | PMMA_50_-*b*-PLMA_100_ | 40 | >99 | 24 600 | 1.13 | Turbid Gel | 0.87 |
| 24 | PMMA_50_-*b*-PLMA_100_ | 10 | 95 | 22 800 | 1.10 | Slightly turbid viscous liquid | 0.87 |
| 25 | PMMA_50_-*b*-PLMA_150_ | 40 | 98 | 31 800 | 1.14 | Turbid Gel | 0.91 |
| 26 | PMMA_50_-*b*-PLMA_150_ | 25 | 86 | 28 600 | 1.20 | Turbid Gel | 0.91 |
| 27 | PMMA_50_-*b*-PLMA_150_ | 10 | 91 | 28 700 | 1.12 | Phase separation | 0.91 |
| 28 | PMMA_50_-*b*-PLMA_200_ | 40 | 92 | 37 900 | 1.28 | Phase separation | 0.93 |

**Table S1.** PMMA*-b-*PLMA Diblock Copolymers Prepared by RAFT-PISA in DGEBA.

^a^:Volume fraction


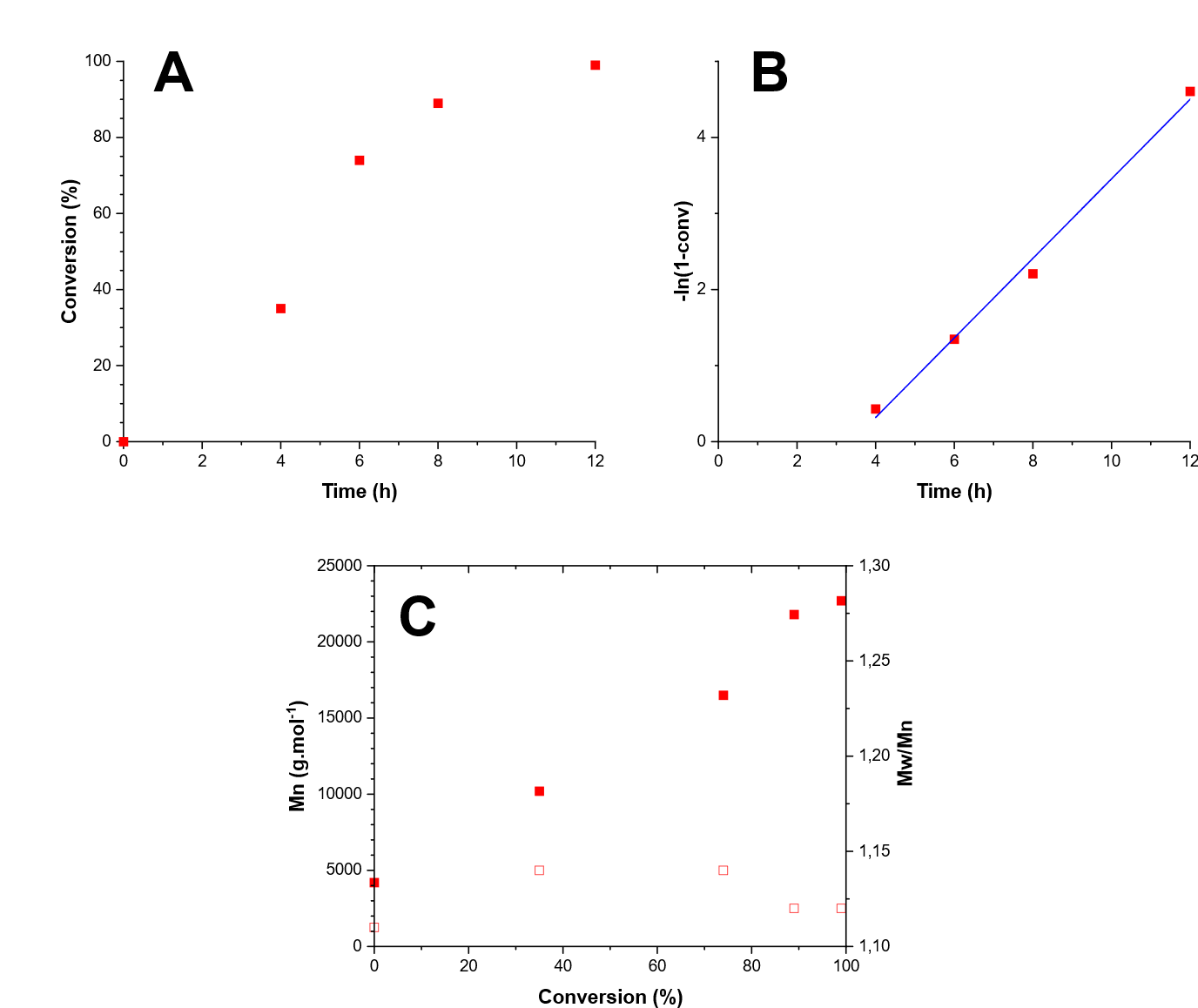


**Figure S1.** Kinetic study of the synthesis of PLMA₅₀-b-PLMA₁₀₀ by RAFT-PISA at 70 °C and 40% w/w solids content in DGEBA (Entry 23, Table S1), using a macro-CTA concentration of 0.0124 mol·L⁻¹ and a feed ratio of [LMA]/[macro-CTA]/[AIBN] = 100/1/0.33. (A) Conversion as a function of time; (B) Pseudo first-order kinetic plots obtained by ^1^H NMR (–ln(1–conversion) vs. time); (C) Evolution of number-average molar mass (Mn) and dispersity (*Đ*) with conversion.

**Figure S2.** SEC traces of the PMMA_50_-*b*-PLMA_y_ BCPs generated by RAFT-PISA at 10% w/w solids content in DGEBA (from right to left, y=0, 25, 50 and 100; entries 18, 20, and 24 in Table

S1).

**Figure S3.** SEC traces of the PMMA_15_-*b*-PLMA_y_ BCPs generated by RAFT-PISA at 10 and 40% w/w solids content in DGEBA (entries 1, 2, 3, 4 and 5 in Table S1).

**Figure S4.** SEC traces of the PMMA_25_-*b*-PLMA_y_ BCPs generated by RAFT-PISA at 40 %w/w solids content in DGEBA (entries 8 and 9 in Table S1).


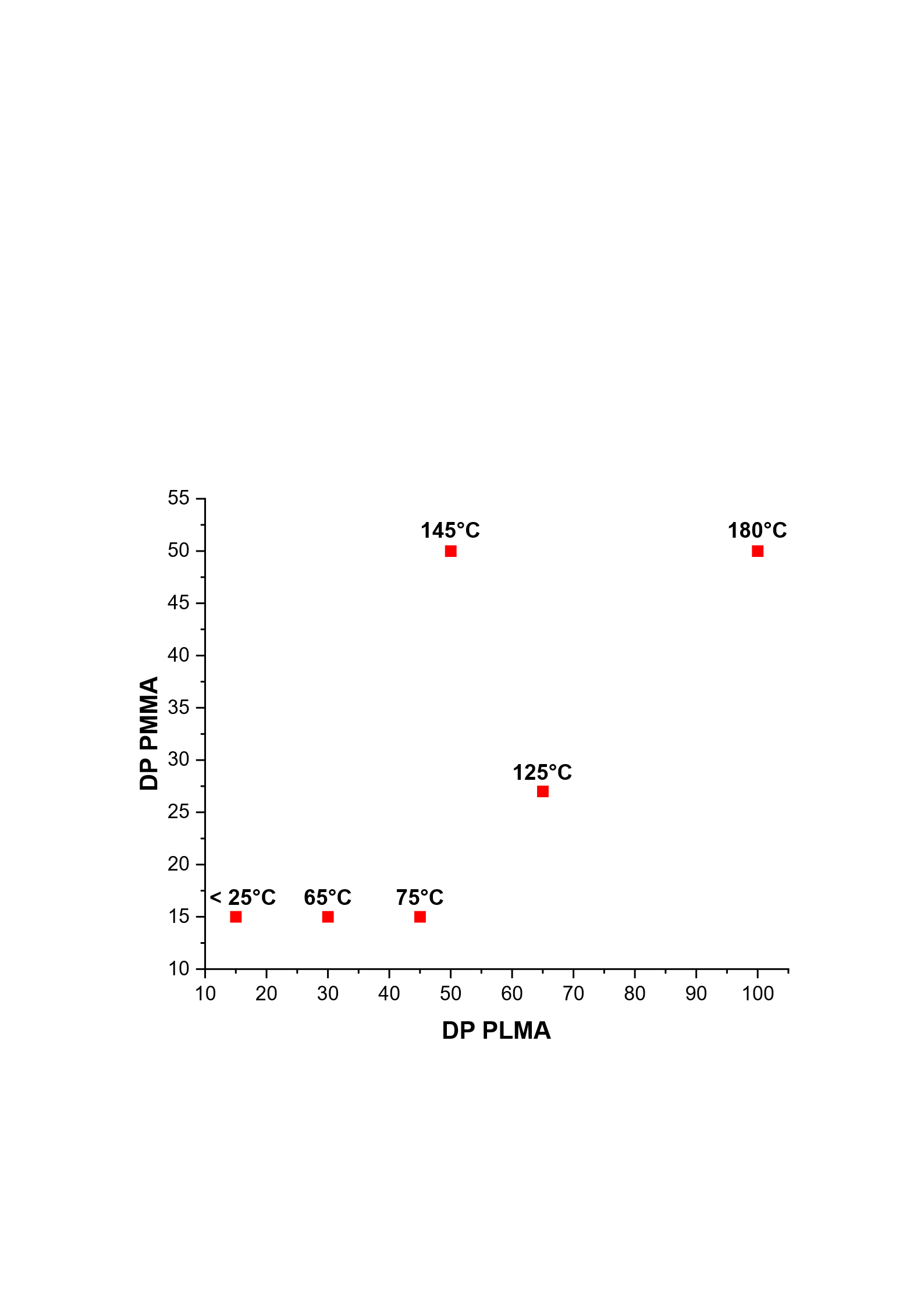


**Figure S5.** Influence of the DP_n_ of PMMA and PLMA blocks on T_ODT_ at 40 wt% in DGEBA as determined by oscillatory rheology (1 Hz, 3 °C/min).

**Figure S6.** SAXS patterns of PMMA_50_-*b*-PLMA_y_ dispersions synthesized at 10% w/w solids content in DGEBA. Lines correspond to the data fits (y=15 or 20: sphere model; y=100: vesicle model).


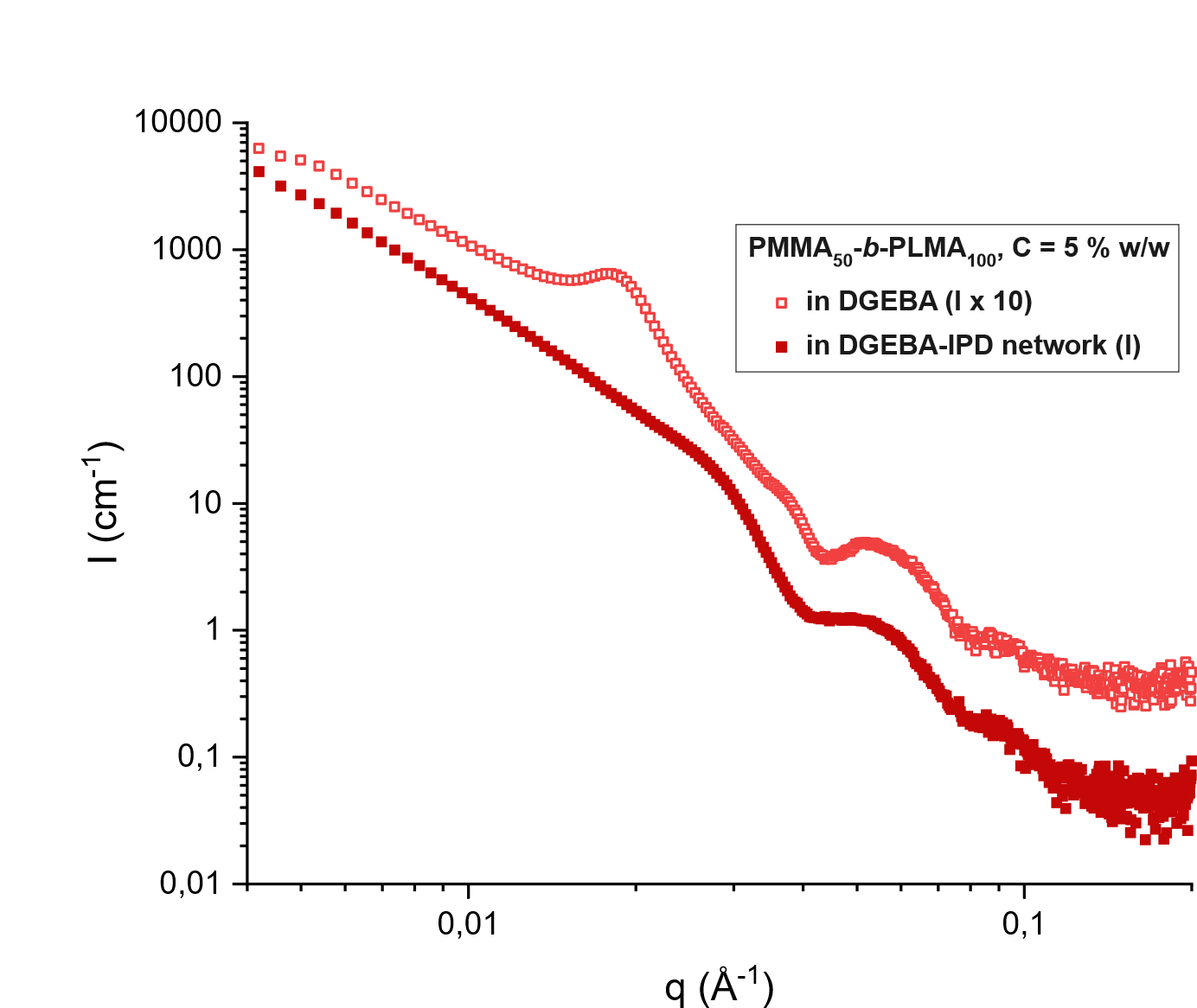


**Figure S7.** SAXS patterns of PMMA_50_-*b*-PLMA_100_ dispersion (generated by PISA at 40% w/w solids content) in DGEBA (diluted at 5% w/w) and resulting DGEBA-IPD network.


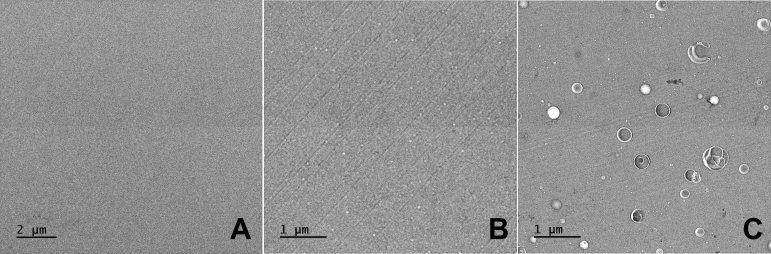


**Figure S8.** TEM pictures of BCP loaded DGEBA-IPD networks. A: TEM picture of neat network B: TEM picture of 1% wt: PMMA_50_-*b*-PLMA_15_ loaded network; C: TEM picture of 1% wt: PMMA_50_-*b*-PLMA_50_ loaded network.

**Figure S9.** Vesicles diameter distribution in ^40^_5_PMMA_50_-*b*-PLMA_100_, ^10^_5_PMMA_50_-*b*-PLMA_100_ and ^40^_5_PMMA_50_-*b*-PLMA_50_ DGEBA-IPD networks.


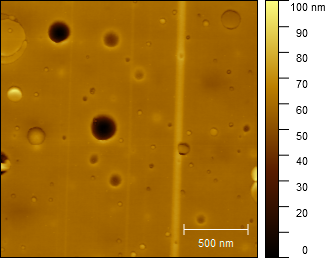


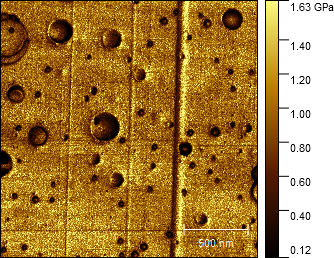


**Figure S10.** Morphology and mechanical contrast of vesicles in 5% w/w: PMMA_50_*-b-*PLMA_50_ loaded-network. Care was taken to ensure that topographical effects do not influence significantly the mechanical characterization of the vesicles; despite some boundary effects, a gradient of elastic modulus is clearly observed.


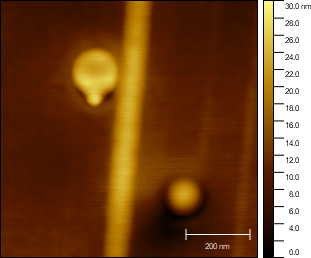

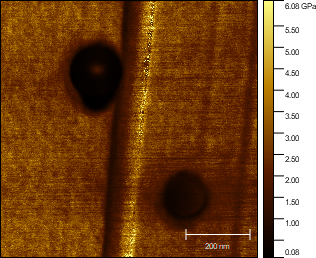


**Figure S11.** Morphology and mechanical contrast of vesicles in 5% w/w: PMMA_50_*-b-*PLMA_100_ loaded-network.

**Figure S12.** Load-displacement curves (1 mm/min, T=25°C) of the reference BCP free networks and BCP loaded networks


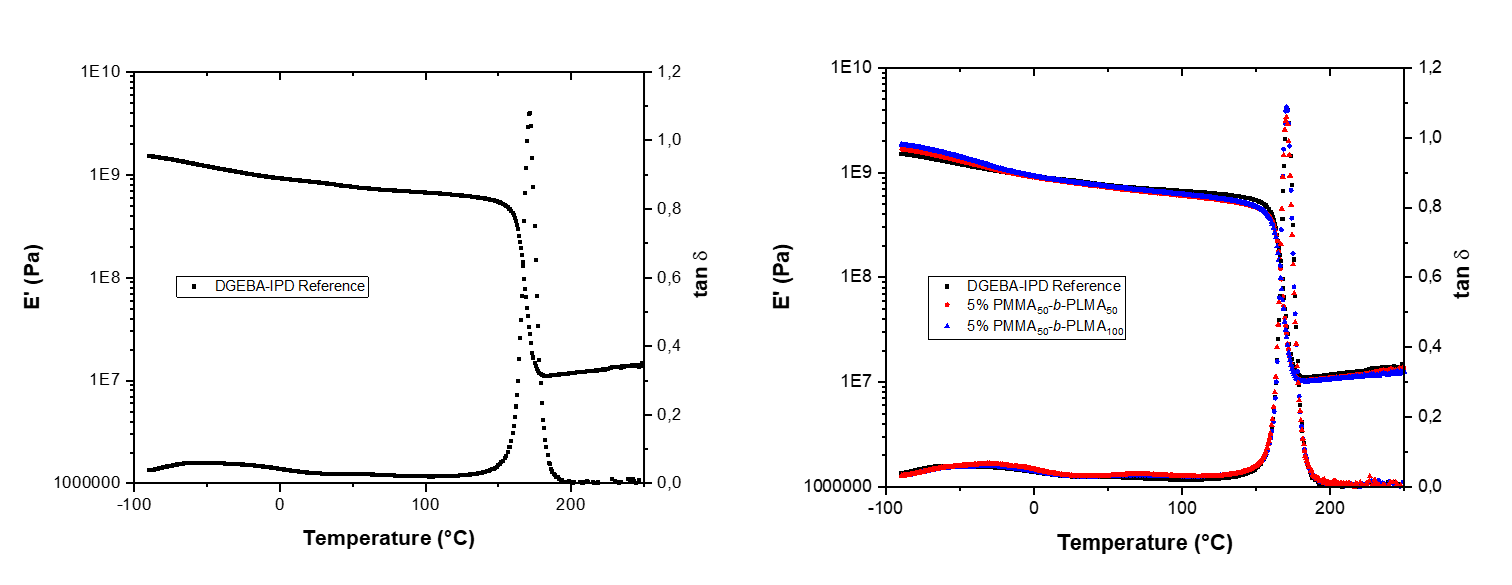


**Figure S13.** Storage modulus and tan δ curves from DMA analyses (f=1 Hz) of the reference DGEBA-IPD network and DGEBA-IPD networks modified with 5% wt of PMMA_50_-*b*-PLMA_50_ or PMMA_50_-*b*-PLMA_100_.

| **No.** | **DGEBA-IPD network** | **w_PLMA_** | **T_α_** | **E’ (25°C) (Pa)** | ***K*_IC_ (MPa.m^1/2^)** |
| --- | --- | --- | --- | --- | --- |
| 1 | Neat | 0% | 172°C | 8.39x10^8^ | 0.59 ± 0.02 |
| 2 | ^40^_5_PMMA_50_-*b*-PLMA_15_ | 2.15% | 164°C | 8.11x10^8^ | 0.79 ± 0.03 |
| 3 | ^40^_1_PMMA_50_-*b*-PLMA_15_ | 0.43% | 168°C | 6.54x10^8^ | 0.60 ± 0.03 |
| 4 | ^40^_5_PMMA_50_-*b*-PLMA_25_ | 2.80% | 166°C | 5.52x10^8^ | 0.72 ± 0.03 |
| 5 | ^10^_1_PMMA_50_-*b*-PLMA_25_ | 0.56% | 167°C | 4.49x10^8^ | 0.75 ± 0.05 |
| 6 | ^40^_5_PMMA_50_-*b*-PLMA_50_ | 3.51% | 171°C | 8.06x10^8^ | 0.85 ± 0.04 |
| 7 | ^40^_1_PMMA_50_-*b*-PLMA_50_ | 0.7% | 168°C | 6.91x10^8^ | 0.86 ± 0.09 |
| 8 | ^10^_1_PMMA_50_-*b*-PLMA_50_ | 0.7% | 169°C | 7.82x10^8^ | 0.72 ± 0.03 |
| 9 | ^40^_5_PMMA_50_-*b*-PLMA_100_ | 4.14% | 171°C | 8.14x10^8^ | 1.05 ± 0.05 |
| 10 | ^10^_5_PMMA_50_-*b*-PLMA_100_ | 4.14% | 166°C | 7.66x10^8^ | 0.86 ± 0.05 |

**Table S2.** Summary of thermomechanical and fracture properties of DGEBA-IPD networks containing various PMMA-*b*-PLMA copolymers.

**Figure S14.** SEC traces of the PMMA_50_-*b*-PLMA_y_ BCPs generated by RAFT-PISA at 40 %w/w solids content in ECC (from right to left, y=0, 50, 75 and 100).

**Figure S15.** SAXS patterns of PMMA_50_-*b*-PLMA_y_ dispersions synthesized at 40% w/w solids content in ECC and diluted at 10% w/w.


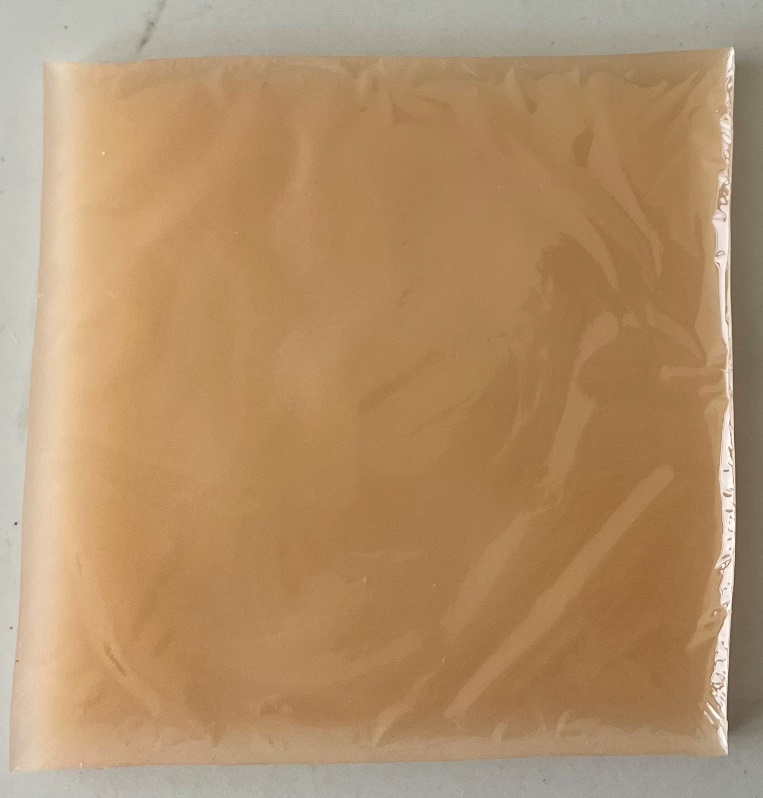


**Figure S16.** Picture of an ECC-based network (2.5 mm thick) containing 1 wt% of PMMA_50_-*b*-PLMA_75_, after photopolymerization and post-curing at 100 °C.
